# Targeting neddylation E2s: a novel therapeutic strategy in cancer

**DOI:** 10.1186/s13045-021-01070-w

**Published:** 2021-04-07

**Authors:** Yi-Chao Zheng, Yan-Jia Guo, Bo Wang, Chong Wang, M. A. A. Mamun, Ya Gao, Hong-Min Liu

**Affiliations:** 1 State Key Laboratory of Esophageal Cancer Prevention and Treatment, Key Laboratory of Advanced Drug Preparation Technologies, Ministry of Education of China, Key Laboratory of Henan Province for Drug Quality and Evaluation, Institute of Drug Discovery and Development, School of Pharmaceutical Sciences, Zhengzhou University, 100 Kexue Avenue, Zhengzhou, 450001 Henan China; 2 Department of Hematology, The First Affiliated Hospital of Zhengzhou University, Zhengzhou, China

**Keywords:** Neddylation, UBE2M, UBE2F, Therapeutic targets, Anticancer treatment

## Abstract

Ubiquitin-conjugating enzyme E2 M (UBE2M) and ubiquitin-conjugating enzyme E2 F (UBE2F) are the two NEDD8-conjugating enzymes of the neddylation pathway that take part in posttranslational modification and change the activity of target proteins. The activity of E2 enzymes requires both a 26-residue N-terminal docking peptide and a conserved E2 catalytic core domain, which is the basis for the transfer of neural precursor cell-expressed developmentally downregulated 8 (NEDD8). By recruiting E3 ligases and targeting cullin and non-cullin substrates, UBE2M and UBE2F play diverse biological roles. Currently, there are several inhibitors that target the UBE2M-defective in cullin neddylation protein 1 (DCN1) interaction to treat cancer. As described above, this review provides insights into the mechanism of UBE2M and UBE2F and emphasizes these two E2 enzymes as appealing therapeutic targets for the treatment of cancers.

## Introduction

NEDD8 is an ubiquitin-like polypeptide with extensive sequence identity (60%) and homology (80%) with ubiquitin [1–5]. Structurally, NEDD8 exhibits four β-sheets characterized by one α-helix and two 3_10_ helices [1, 6] and has two domains: a flexible carboxy-terminal tail domain and a globular ubiquitin-fold domain (UFD). The Gly–Gly sequence in the tail end conjugates to target proteins and uses different extended structures to combine with neddylation and deneddylation enzymes [7–12].

**Fig. 1:**
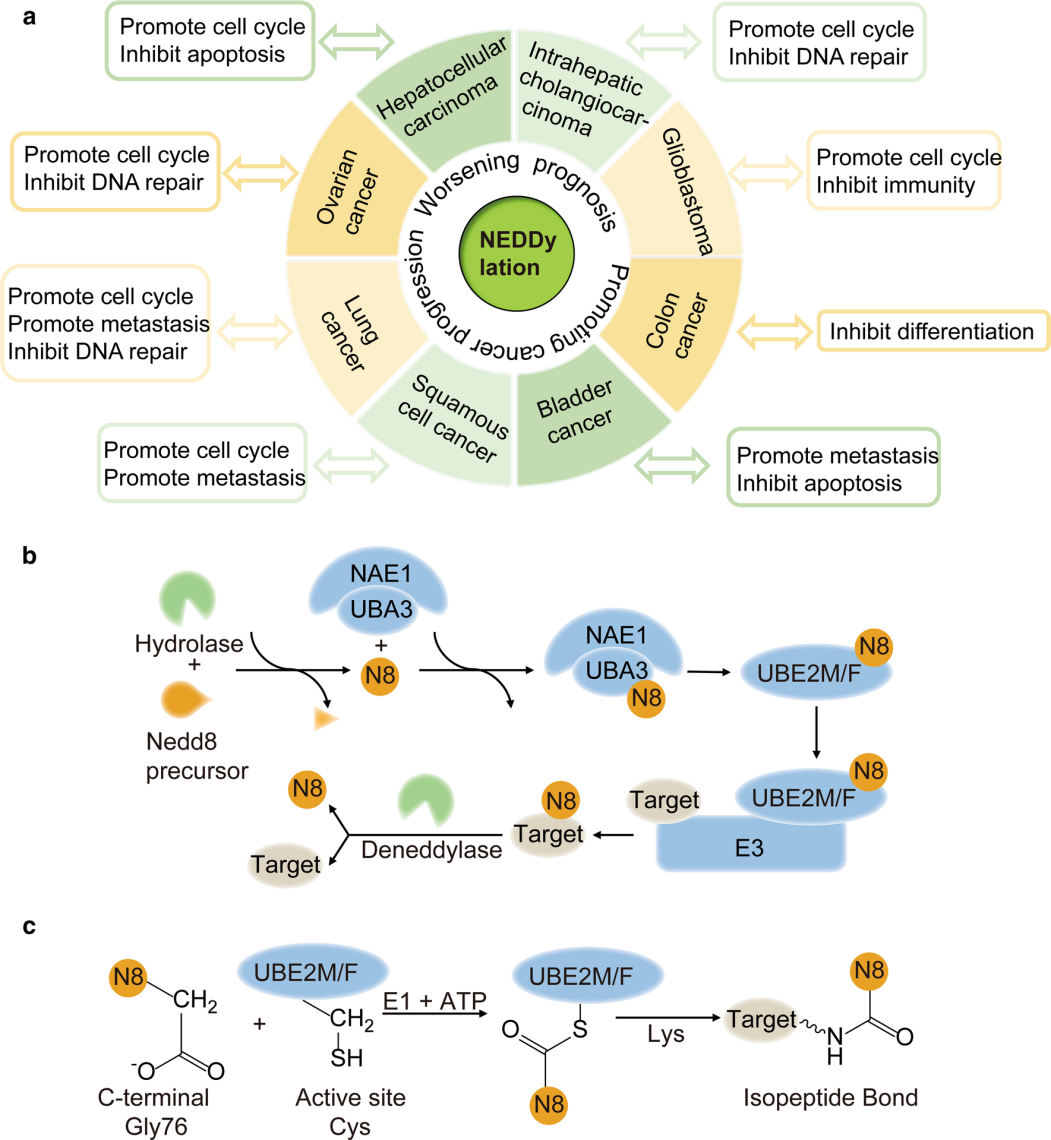
Overview of the neddylation pathway. **a** Impact of neddylation in cancers. **b** The process by which NEDD8 is conjugated to its substrates. NEDD8 is activated by an NAE in an ATP-dependent manner, transferred to E2 and then conjugated to a lysine residue of the substrate protein with the aid of E3 ligase. Then, NEDD8 is removed by a deneddylase from the substrate and recycled. **c** The C-terminal carboxylate of NEDD8 is conjugated to the active cysteine residue of the E2 enzyme in the catalysis of E1. The E2-NEDD8 conjugate reacts with a lysine residue on the substrate to form the NEDD8 linkage

Protein neddylation, which involves transfer of NEDD8 to a lysine residue of the substrate, is a process that changes the substrate's activity, conformation, and subcellular localization, not for degradation [13–16]. In many cancers, overactivation of the neddylation pathway causes an increase in the levels of tumor-promoting factors and a decrease in the levels of tumor suppressors, thereby promoting the occurrence of tumors and worsening prognosis (Fig. 1a) [17–24]. In mammalian cells, like ubiquitylation, neddylation starts with ATP-dependent activation of the NEDD8 C-terminus by an E1 NEDD8-activating enzyme (NAE) resulting in the formation a thioester-linked E1-NEDD8 complex. The NAE consists of a heterodimer of NAE1 (APPBP1) and ubiquitin-like modifier activating enzyme 3 (UBA3, NAEβ) subunits [9, 25–28]. Then, activated NEDD8 is transferred to a NEDD8-conjugating enzyme (E2), including the well-studied enzyme UBE2M (also known as UBC12) and the less characterized enzyme UBE2F [29–32], to form another thioester via a transthiolation reaction. Finally, a NEDD8 E3 ligase that binds both the E2-NEDD8 complex and the substrate transfers NEDD8 over to the ε-amino group of the lysine residue in the target protein to form an isopeptide bond. In light of their mechanistic strategy, the majority of the NEDD8 E3 ligases contain interesting novel gene (RING) finger domains, including RING-box protein 1 (RBX1, ROC1) [33–36], RING-box protein 2 (RBX2, ROC2) [29, 37–42], murine double minute 2 (MDM2) [43–45], casitas B-lineage lymphoma (c-CBL) [46–49], F-box protein 11 (FBXO11) [50–52], inhibitor of apoptosis (IAP) [53–55], RNA polymerase II transcription factor B subunit 3 (TFB3) [56], tripartite motif 40 (TRIM40) [57], ring finger protein 168 (RNF168) [58], and ring finger protein 111 (RNF111) [59, 60] domains. Interestingly, DCN1, a protein conserved from yeast to mammals, is also a NEDD8 E3 ligase but retains its catalytic activity despite not containing a RING finger domain [61–64]. Furthermore, neddylated substrates can be deneddylated by deneddylases, such as NEDD8 protease 1 (NEDP1) [7, 65–69] and COP9 signalosome (CSN) [70–76]. Hence, neddylation is a reversible process, as NEDD8 can be recycled [2, 11, 77–79] (Fig. 1b).

The neddylation E2 enzymes UBE2M and UBE2F are only twice as large as NEDD8, and they primarily take part in two types of reactions: transthiolation-transfer from a thioester to a thiol group and aminolysis-transfer from a thioester to an amino group (Fig. 1c). By cooperating with E1 and other E3 enzymes, the E2 enzymes are specific for NEDD8 in catalysis of the neddylation reaction [65, 80]. Therefore, the two E2 enzymes are central players in this enzymatic reaction, in addition to being carriers of NEDD8.

This review discusses the structure of neddylation-related E2 enzymes and summarizes the current understanding of their mechanism and effect in biological processes. UBE2M and UBE2F may become promising targets for cancer treatment.

## Structure and basic biology of UBE2M and UBE2F

### UBE2M

**Fig. 2:**
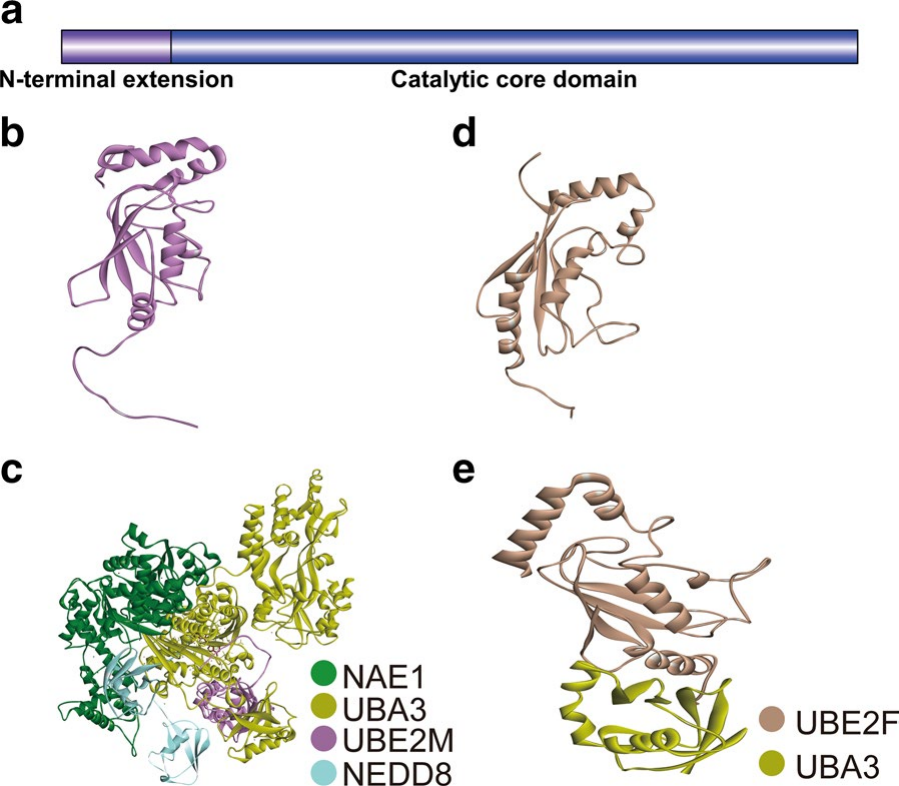
Structural analysis of UBE2M and UBE2F. **a** Primary sequence map of UBE2M and UBE2F. **b** Structure of UBE2M (PDB 2NVU). **c** Structure of the NAE-UBE2M complex. The NAE contains NAE1 and UBA3 (PDB 2NVU). **d** Structure of UBE2F (PDB 2EDI). **e** Structure of the UBA3 and UBE2F complex (PDB 3FN1)

Full-length UBE2M (human) includes 183 amino acids and consists of two regions: a 26-residue N-terminal docking peptide and an ~ 150-residue conserved E2 catalytic core domain. And UBE2M functions as a unique E2 ubiquitin-conjugating enzyme in neddylation. Unlike E2s, the 26-residue N-terminal docking peptide of UBE2M is specific for the NEDD8 pathway, as UBE2M’s N-terminal docking peptide is conserved across species and cannot be found in other E2s (Fig. 2a, b) [81]. Furthermore, crystallographic studies [9, 82] of APPBP1-UBA3-UBE2M have revealed that the NAE has three domains: (1) an adenylation domain with an ATP-binding site, (2) a domain surrounding the catalytic cysteine, and (3) a C-terminal domain. The E1–E2 interaction occurs in a bipartite manner: UBE2M’s N-terminal peptide and core domain bind to the NAE to complete the transfer of the NEDD8 from E1 to E2 [81, 83, 84]. The E2’s core domain binds to the C-terminal ubiquitin fold domain, and residues 1–13 of UBE2M’s N-terminal extension and cooperates with a big docking groove in the adenylation domain of E1, which is stabilized via many hydrogen bonds. Although the adenylation domain of UBA3 is conserved among ubiquitin-like protein (UBL)-activating enzymes, the E1-E2 interaction is likely to be unique to the NEDD8 pathway since UBE2M’s N-terminal extension is unique (Fig. 2c) [81].

### UBE2F

Full-length human UBE2F includes 185 amino acids and functions as a unique E2 ubiquitin-conjugating enzyme for neddylation. Similar to UBE2M, UBE2F has an N-terminal extension and a conserved catalytic core domain. However, its features are different from those of UBE2M; for example, the N-terminal α1 helix of the UBE2F’s core domain has an offset orientation, the catalytic cysteine is inserted in a loop following the catalytic Cys116 residue, and the C-terminal extension has an α helix instead of a two-stranded β sheet (Fig. 2a, d) [29]. The interaction between UBE2F and the NAE bears great similarity to the UBE2M and NAE interaction: both the N-terminal extension and core domain bind to the NAE. However, the sequence of UBE2M’s N-terminal extension that binds to the UBA3 docking groove is Leu4-Phe5-X-Leu7, whereas the interaction between the UBE2F’s N-terminal extension and UBA3 is through the sequence Met-Leu2-X-Leu4 (Fig. 2e) [29].

## Relationship between UBE2M and UBE2F

UBE2M and UBE2F, which serve as two E2 enzymes, transfer NEDD8 to NEDD8 E3 ligases. Structurally, they are similar to each other and can combine with other enzymes in the same catalytic manner. They both play key roles in the neddylation of cullins to activate cullin-RING ligases (CRLs) [10]. However, they are indeed two independent E2 enzymes and display distinct functions [29]. UBE2M couples with RBX1 to induce the neddylation of cullin1, cullin2, cullin3, cullin4A, and cullin4B and triggers corresponding CRLs, which can influence the levels of its substrates, such as p21, p27, Bim, and WD repeat domain phosphoinositide-interacting protein 2 (WIPI2), to take part in autophagy, the cell cycle and DNA repair [29, 85–88]. UBE2F can pair with RBX2 to activate the ligase CRL5 by forming a UBE2F/RBX2/cullin5 complex, leading to the enrichment of NOXA, thereby participating in apoptosis progression and inhibiting cancer cell growth [89]. UBE2M shows intrinsic specificity for RBX1, but RBX2 shows an intrinsic specificity for UBE2F [3, 29]. Notably, MLN4924 [90–95], which is also known as pevonedistat, is a first-in-class inhibitor of the NAE used in cancer treatment and regulates UBE2M and UBE2F in different ways; MLN4924 causes a dose- and time-dependent increase in UBE2M levels but a decrease in UBE2F levels [89].

**Fig. 3:**
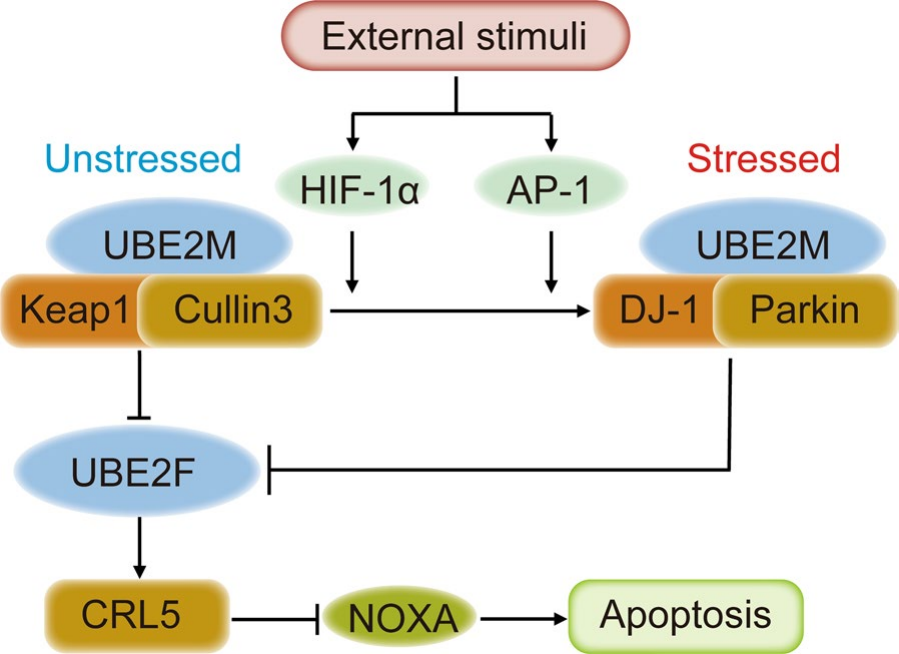
Cross-talk between the E2 and E3 [89]. UBE2M, a stress-inducible protein promoted by HIF-1α and AP-1, plays a dual role as an E2 for ubiquitylation and neddylation to degrade the UBE2F, leading to the inactivation of CRL5 mediated by CRL3

In addition, Zhou et al. [96] found that UBE2M is a stress-inducible protein that can be promoted by hypoxia-inducible factor 1α (HIF-1α) and transcription factor AP-1 (AP-1) and plays a dual role as an E2 for ubiquitylation and neddylation to degrade UBE2F (Fig. 3). Under normal physiological conditions, UBE2M serves as a neddylation E2 that can couple with cullin3 and Kelch-like ECH-associated Protein 1 (Keap1) to trigger UBE2F polyubiquitylation and degradation. However, under stressed conditions, UBE2M forms a novel E2–E3 complex, UBE2M/DJ-1/Parkin (DJ-1, also known as Parkinson disease protein 7; Parkin, E3 ubiquitin-protein ligase parkin), with the inducement by HIF-1α and AP-1, and promotes the ubiquitylation and degradation of UBE2F. Then, CRL5 is deactivated, and the substrate NOXA can be enriched, leading to the promotion of apoptosis and cell growth inhibition of lung cancer cells. Taken together, these findings indicate that UBE2M can decrease the amount of UBE2F via two E3 ligases, leading to the inactivation of CRL5 mediated by CRL3, which demonstrates cross-talk between the E2 and E3.

## UBE2M and UBE2F in cancer

UBE2M and UBE2F play crucial roles in various biological processes by recruiting E3 ligases and targeting the substrates of cullins and non-cullins. Several studies have revealed that UBE2M and UBE2F are both overexpressed in multiple types of cancers, such as hepatocellular carcinoma, lung adenocarcinoma, osteosarcoma, ovarian cancer, and squamous cell carcinoma (Table 1) [97–101], and bioinformatics analysis of The Cancer Genome Atlas (TCGA) datasets has revealed that their expression is upregulated in cancer tissues compared with normal tissues [102–104]. They both act as oncogenes by promoting the neddylation of specific substrates to regulate diverse signaling pathways, regulating several cell biological processes, such as DNA repair, genomic stability, apoptosis, autophagy, and the cell cycle.

**Table 1:** Expression and clinical significance of UBE2F and UBE2M in tumors

	Cancer	Expression	Function
**UBE2M**	Osteosarcoma [98]	Overexpressed	Promotes cell viability
	Hepatocellular carcinoma [19, 86]	Overexpressed	Associated with poor prognosis
	Lung cancer [87]	Overexpressed	Associated with poor survival
	Breast cancer [99]	Overexpressed	Worsens prognosis
	Intrahepatic cholangiocarcinoma [101]	Overexpressed	Associated with prognosis
	Osteoarthritis [100]	Overexpressed	Promotes apoptosis
	Esophageal squamous cell carcinoma [88]	Overexpressed	Associated with poor survival
**UBE2F**	Non-small cell lung cancer [89]	Overexpressed	Associated with poor survival

### Apoptosis and autophagy

**Fig. 4:**
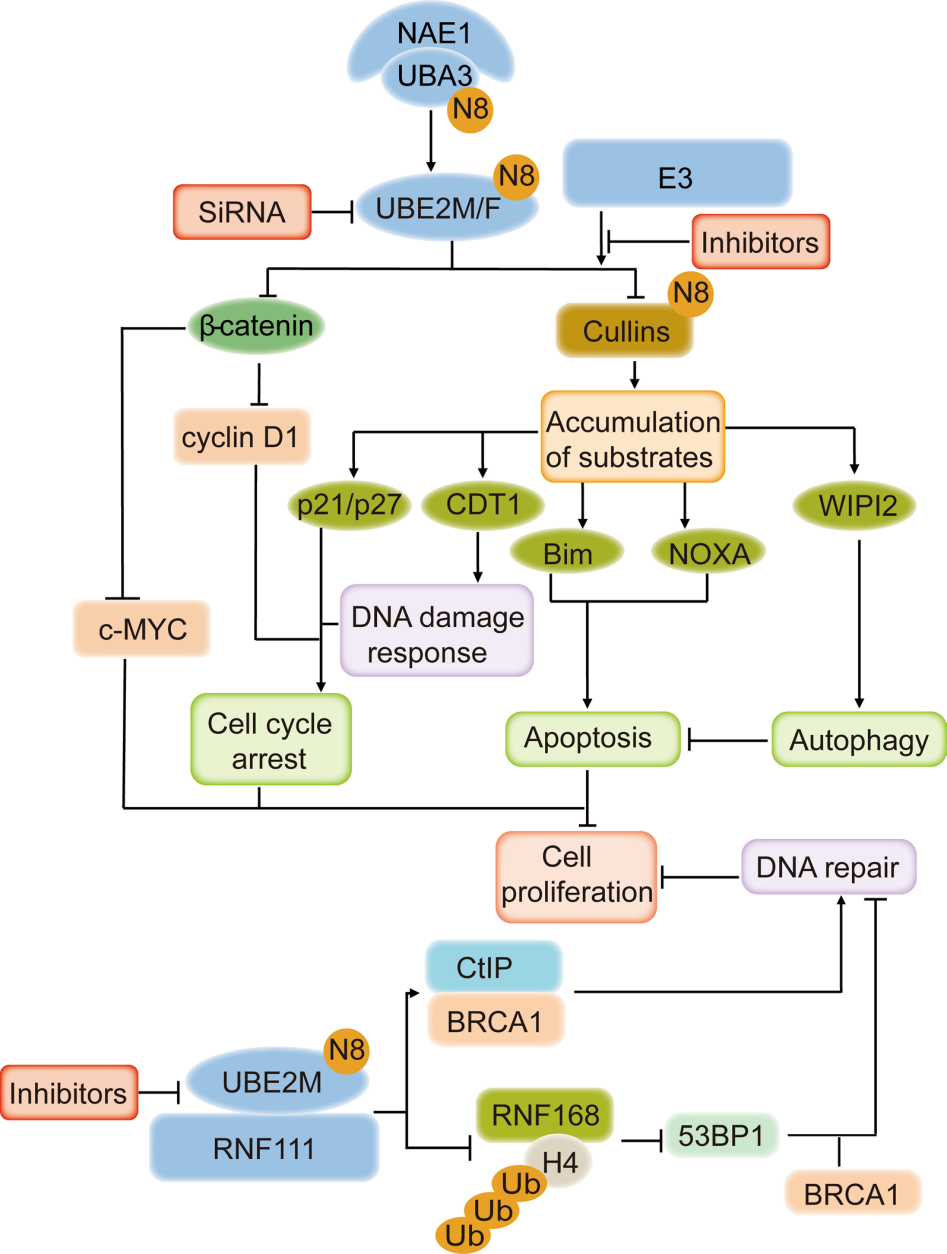
UBE2M and UBE2F in cancer. Inhibiting the activity of UBE2M and UBE2F can inhibit cell proliferation. UBE2M can promote cell cycle progression to induce cell growth by stabilizing β-catenin and activating the neddylation of cullin1-4. UBE2M can recruit RNF111 to disturb the CtIP-BRCA1 interaction and promote H4 protein ubiquitylation via the neddylation of RNF168 to regulate DNA repair. UBE2F can reduce the level of NOXA by triggering CRL5 and then inhibit apoptosis and induce cell growth

UBE2M and UBE2F play an essential role in apoptosis and autophagy-mediated by CRLs. UBE2M couples with RBX1 and damage-specific DNA-binding protein 1 (DDB1) to induce cullin4A neddylation and trigger the ubiquitin ligase CRL4A, which can induce the ubiquitination and degradation of WIPI2, an autophagy-associated protein, resulting in cell proliferation [105]. Thus, knockdown of UBE2M can block the autophagy process and inhibit cell proliferation [105]. UBE2F can also take part in the cell apoptosis pathway by downregulating the expression of the proapoptotic protein NOXA [89]. By recruiting RBX2, UBE2F can trigger the neddylation and activation of cullin5, promoting the ubiquitylation and degradation of its substrate NOXA. Knockdown of UBE2F or its mutant (C116A) can induce the accumulation of NOXA, inducing apoptosis of lung cancer cells (Fig. 4).

### Cell cycle

In cancer cells, the expression of cell cycle inhibitor proteins is usually downregulated, whereas proteins that promote cell cycle progression are generally overexpressed. Rescuing the levels of cell cycle inhibitor proteins is the primary strategy for disrupting cell cycle progression [106–108]. As UBE2M and UBE2F have been reported to take part in the progression of the cell cycle, E2 enzyme abrogation can arrest cell cycle progression and inhibit cell growth [86, 87]. In lung cancer, knockdown of UBE2M can promote the expression of cyclin-dependent kinase inhibitor 1 (CDKN1A and CDKN1B) and cyclin-associated proteins (such as G2/mitotic-specific cyclin-B1, Cyclin-A2, G1/S-specific cyclin-D3, and Cyclin-dependent kinase 4 homolog) in cell cycle progression. Then, the cell cycle can be arrested at G2 phase and fail to progress to M phase [87]. In hepatocellular carcinoma (HCC), UBE2M can stabilize β-catenin and increase the level of its downstream protein cyclin D1 to promote the progression from G0/G1 phase to S phase (Fig. 4) [86].

### DNA damage response

Neddylation can also contribute to the DNA damage response [58, 59]. For example, UBE2M participates in DNA damage repair and maintains genomic stability via multiple CRLs [85, 101]. As UBE2M can promote the neddylation of cullins and then activate CRLs, the abrogation of UBE2M expression leads to DNA double-strand breaks (DSBs) and increases cell sensitivity to DNA-damaging agents. Scott et al. found that knockdown of UBE2M leads to the blockage of cell cycle progression from G1 to S phase and is related to a delay in the S-phase-dependent DNA damage response [85]. Moreover, UBE2M expression abrogation can also attenuate nonhomologous end-joining (NHEJ) and inhibit cell proliferation [109].

In addition, UBE2M can promote DNA repair via non-cullins. RNF111 together with UBE2M can promote the neddylation of RNF168 and thereby induce the ubiquitylation of histone H4, leading to the activation of DNA repair via 53BP1 and breast cancer susceptibility gene 1 (BRCA1) [59]. It is worth mentioning that UBE2M/RNF111-induced neddylation blocks the interaction between CtIP and BRCA1 and then inhibits the CtIP and BRCA1-mediated DNA end resection process, an essential process in the repair pathway [110]. Coincidentally, RNAi-mediated knockdown of UBE2M sensitizes the hormone-resistant prostate cancer cell line DU145 to radiation-induced DSBs [111] (Fig. 4).

## Inhibitors of UBE2M

In various cancers, such as hepatocellular carcinoma, lung adenocarcinoma, ovarian cancer, and osteosarcoma, neddylation is always overactivated [13, 15, 19, 78, 112–114]. MLN4924 is an inhibitor of the NAE and used as an anticancer drug [10, 94, 115, 116]. To date, many studies revealed that MLN4924 plays a significant role in cell cycle arrest and the DNA damage response, inhibiting angiogenesis and tumor growth and inducing apoptosis, autophagy, and senescence [117–119]. Although 38 clinical trials have been performed and five completed phase I clinical trials demonstrated that MLN4924 is safe and feasible to date, there is an issue with the specificity of MLN4924, and cancer cells can develop resistance to MLN4924 [120–122].

**Fig. 5:**
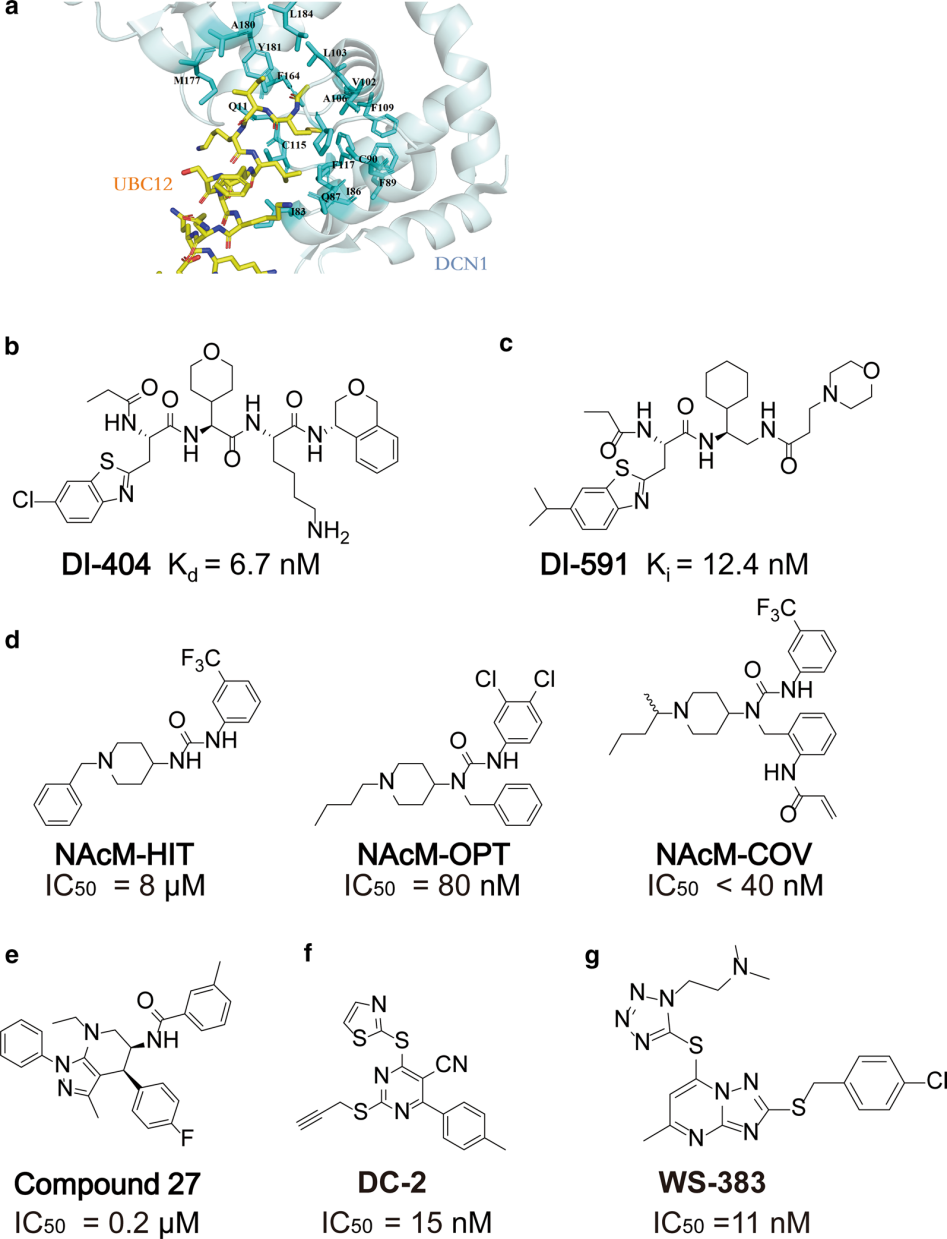
UBE2M-DCN1 inhibitors. **a** The crystal structure of the interaction between DCN1 and the UBE2M peptide is shown in yellow (PDB 3TDU). **b**–**g** Chemical structures of UBE2M-DCN1 inhibitors

To overcome the limitations of MLN4924, some studies have focused on discovering specific inhibitors against neddylation-related E2s to achieve more specific modulation of cullin neddylation. Hence, the development of UBE2M-DCN1 protein–protein interaction inhibitors was pursued by medicinal chemists due to the druggable interaction between UBE2M and DCN1 (Fig. 5a; Table 2) [123].

**Table 2:** Summary of inhibitors targeting UBE2M and DCN1

Compound	IC_50_ (TR-FRET)	Effect	Cell line	Clinical trial
**DI-404 **[125]	N/A	Inhibits the neddylation of cullin3	Lung cancer cells	N/A
**DI-591 **[124]	N/A	Inhibits the neddylation of cullin3 and increases the expression of NRF2	Liver cells	N/A
**NAcM-HIT **[129]	8 μM	Disturbs the interaction between UBE2M and DCN1	Lung cancer cells	N/A
**NAcM-OPT **[130]	80 nM	Inhibits the neddylation of cullin3 and cullin1	Liver cells	N/A
**NAcM-COV **[131]	< 40 nM	Reduces the steady-state levels of neddylated cullin1 and cullin3	Liver cells	N/A
**DC-2 **[132]	15 nM	Decreases the neddylation of cullin3	Lung cancer cells	N/A
			Esophageal cancer cells	
			Liver cancer cells	
			Breast cancer cells	
			Prostatic cancer cells	
**Compound 27 **[133]	0.2 μM	Disturbs the neddylation of cullin1 and cullin3	Lung cancer cells	N/A
**WS-383 **[134]	11 nM	Inhibits the neddylation of cullin1 and cullin3	Gastric cancer cells	N/A

To discover UBE2M-DCN1 inhibitors, Zhou and colleagues designed potent peptidomimetics by extensively modifying the N-terminal 12-residue peptide of UBE2M, such as **DI-404** (Fig. 5b) [124] and **DI-591** (Fig. 5c) [125]. At the biochemical level, **DI-404** exhibited a high affinity for DCN1 with a K_d_ value of 6.7 nM and good solubility of 54 µM in PBS at pH 7.4. DCN1 and RBX1 act as co-E3s to promote cullin neddylation [126–128]. A cellular study suggested that **DI-404** cannot regulate the levels of UBE2M and DCN1 but can reduce the association between UBE2M and DCN1. Notably, **DI-404** can selectively inhibit the neddylation of cullin3 but not the neddylation of other cullins [125]. Due to the peptidic nature of **DI-404**, although **DI-404** showed a high binding affinity for DCN1, it only exhibited moderate cellular activity. Thus, to address this problem and obtain more drug-like compounds, Zhou et al. designed **DI-591** through a series of structure-based optimizations [125]. At the biochemical level, **DI-591** can bind DCN1 with a *K*_*i*_ value of 12.4 nM and a *K*_*d*_ value of 30.6 nM, as **DI-591** uses its bicyclic ring to interact with the subpocket of DCN1, exhibiting extensive hydrophobicity. In addition, the propionyl group and cyclohexyl group are crucial in forming hydrophobic interactions with DCN1. **DI-591**, which selectively inhibits the neddylation of cullin3 in a dose-dependent manner, shows a remarkable similarity to **DI-404**. Thus, **DI-591** can increase the expression of nuclear factor erythroid 2-related factor 2 (NRF2), the substrate of CRL1 and CRL3, but fails to increase the levels of p21 and Bim, the substrate of CRL1. In addition, **DI-591** displayed no cytotoxicity in THLE2 human liver epithelial cells [124].

Moreover, Guy and Schulman’s group identified nonpeptidic and potent small molecule UBE2M-DCN1 inhibitors from over 600,000 compounds through a time-resolved fluorescence energy transfer (TR-FRET) assay (Fig. 5d) [129–131]. One of the identified molecules, **NAcM-HIT**, can be docked in UBE2M’s *N*-acetyl-Met-binding pocket in DCN1, breaking the interaction between UBE2M and DCN1. Based on the optimization of **NAcM-HIT**, an inhibitor, **NAcM-OPT**, was designed, which displays 100-fold better potency than **NAcM-HIT**. **NAcM-OPT** can selectively reduce cullin1 and cullin3 neddylation by binding DCN1 and DCN2 in cells and induce the expression of the two known substrates of CRLs, NRF2 and p21. Moreover, **NAcM-OPT** has strong potential in vivo because of its stability and oral bioavailability. In addition, **NAcM-COV** is another inhibitor that was optimized from **NAcM-HIT** and can bind to DCN1 irreversibly by targeting the Cys115 residue of DCN1. However, **NAcM-COV** cannot interact with DCN5 because of its lack a cystine residue corresponding to the Cys115 residue of DCN1, emphasizing the critical role of the Cys115 residue. Consistently, treatment with **NAcM-COV** reduced the steady-state levels of neddylated cullin1 and cullin3 and enriched their substrates, which can inhibit cell growth. However, due to several disadvantages of **NAcM-like** inhibitors, including (1) their lack of a three-dimensional character due to the presence of only one stereocenter and (2) the need for frequent and high doses to maintain appropriate concentrations in mouse models due to their moderate half-lives [129–131], Guy’s research group [133] designed and synthesized **compound 27** based on the structure of pyrazolopyridone (Fig. 5e). The researchers suggested that it has a greater degree of three-dimensional structure owing to its two chiral centers, allowing it to more easily dock into the binding pocket of DCN1. Thus, **compound 27** is more potent than **NAcM-like** inhibitors and can engage cellular DCN1 and selectively decrease the neddylation of cullin1 and cullin3.

In addition, Zhou et al. identified compound **DC-1** from 1000 compounds [132]. Then, via an extensive structure–activity relationship (SAR) study, a novel small molecule inhibitor, **DC-2**, was discovered. **DC-2** (Fig. 5f) can inhibit the interaction between UBE2M and DCN1 and disturb the neddylation of cullin3. Wang et al. obtained compound **WS-383** by screening and optimizing a series of compounds [134]. **WS-383** (Fig. 5g), a triazolo[1,5-α] pyrimidine-based inhibitor targeting the UBE2M-DCN1 interaction, selectively inhibits the neddylation of cullin1 and cullin3 and increases the expression of p21, p27, and NRF2. All these compounds provide guidance to identify more potent UBE2M-DCN1 protein–protein interaction inhibitors.

## Targeting E2 enzyme for anticancer therapy

The data from preclinical trials and clinical research [14, 92] have revealed the potency, activity, and effectiveness of **MLN4924**, indicating that the neddylation pathway is a potentially powerful therapeutic target. However, resistance can also occur: Mutations in the ATP-binding pocket of UBA3 can inhibit the formation of the **MLN4924**-NEDD8 adduct [120], which reduces the response to it and limits its clinical application. Hence, other targets in the neddylation pathway are urgently needed as alternative strategies for targeting NAE1.

### Targeting UBE2M for anticancer therapy

In recent years, some studies have indicated that UBE2M might be an attractive alternative therapeutic target. By using two available Affymetrix microarray datasets [135, 136], Li et al. [87] identified that UBE2M, but not NAE1 and UBA3, is overexpressed in multiple types of lung cancers and associated with poor survival outcomes, as UBE2M can induce cell proliferation. In contrast, UBE2M knockdown showed a powerful effect in inhibiting tumor growth and metastasis [85–87]. Moreover, the above-mentioned series of inhibitors have been discovered to target the interaction between UBE2M and DCN1, resulting in the inactivation of the ligases CRL1 and CRL3 and the enrichment of their substrates, such as NRF2, p21, and p27. Therefore, these UBE2M-DCN1 inhibitors, which have excellent potencies and pharmacokinetic characteristics, may have therapeutic potential for the treatment of human cancers. In summary, there is enough evidence to prove that UBE2M may be a promising therapeutic target for cancer treatment.

### Targeting UBE2F for anticancer therapy

Another E2 enzyme, UBE2F, has not been studied extensively. However, Zhou et al. [89] found UBE2F has potential as an anticancer target. First, UBE2F is overexpressed in non-small cell lung cancer (NSCLC) and can be used to predict patient survival outcomes. Overexpression of UBE2F promotes NOXA degradation, leading to inhibition of apoptosis and thus increasing cell survival. Thus, targeting the UBE2F/RBX2/CRL5 axis either with small-molecule inhibitors such as MLN4924 or by genetic depletion of either component would inactivate CRL5 to cause NOXA accumulation and apoptosis induction, thus antagonizing UBE2F-mediated growth-stimulating processes. Although there has been only one report about the biological role of UBE2F in cancer and while no inhibitors are available to target UBE2F, these results provide evidence that UBE2F may be a potential and novel target for cancer treatment.

## Conclusion

This review discusses the current knowledge on the neddylation E2 enzymes UBE2M and UBE2F, including their structures, binding partners, substrates, and roles in the diverse biological processes. In addition, it describes how neddylation-related E2 enzymes function in cells under both normal and pathological conditions. Recent and ongoing investigations have proven that UBE2M and UBE2F are overexpressed in cancer cells and associated with cell proliferation and poor survival rates. UBE2M can facilitate cell cycle progression and autophagy by activating CRLs and reducing the levels of their substrates, leading to cell growth [86, 105]; moreover, UBE2M can also take part in NHEJ, and deletion of UBE2M leads to DSBs [59, 85, 101, 109, 110]. Coincidentally, UBE2F in complex with RBX2 can also inhibit cell apoptosis, resulting in cell proliferation [89]. Therefore, small-molecule inhibitors targeting UBE2M-DCN1 have emerged. Moreover, a cellular biological study showed that UBE2M-DCN1 inhibitors may serve as promising compounds for cancer treatment by blocking the neddylation of cullin1 and cullin3 and augmenting the levels of tumor suppressors. Nevertheless, UBE2M-DCN1 inhibitors, which were identified as discussed in this review, have not entered clinical trials, and it is critical to develop more specific and potent inhibitors.

Taken together, UBE2M may be a novel and appealing target, and UBE2F may become a potential target for cancer treatment. Unquestionably, they deserve to be studied further.

## Data Availability

Not applicable.

## Abbreviations

AP-1: Transcription factor AP-1
c-CBL: Casitas B-lineage lymphoma
CDKN1A/B: Cyclin-dependent kinase inhibitor 1
CDT1: DNA replication factor Cdt1
CRLs: Cullin-RING ligases
CSN: COP9 signalosome complex
DDB1: Damage-specific DNA binding protein 1
DSBs: DNA double-strand breaks
HCC: Hepatocellular carcinoma
HIF-1α: Hypoxia-inducible factor 1α
HR: Homologous recombination
IAPs: Inhibitor of apoptosis
LUAD: Lung cancer tissue adenocarcinomas
MDM2: Murine double minute 2
NAE: NEDD8-activating enzyme
NEDD8: Neural precursor cell expressed, developmentally downregulated 8
NEDP1: NEDD8 protease 1
NRF2: Nuclear factor erythroid 2-related factor 2
NSCLC: Non-small cell lung cancer
PTM: Posttranslational modification
RBX1/2: RING-box protein 1/2
RING: Really interesting new gene
RNF111: Ring finger protein
SAR: Structure–activity relationship
TCGA: The Cancer Genome Atlas
TRIM40: Tripartite motif 40
UBA3: Ubiquitin-like modifier activating enzyme 3
UBE2F/M: Ubiquitin-conjugating enzyme E2 F/M
UFD: Ubiquitin-fold domain
UBL: Ubiquitin-like protein
WIPI2: WD repeat domain, phosphoinositide interacting 2

## References

[1] Kamitani T, Kito K, Nguyen HP, Yeh ET (1997). Characterization of NEDD8, a developmentally down-regulated ubiquitin-like protein. J Biol Chem.

[2] Zhou L, Zhang W, Sun Y, Jia L (2018). Protein neddylation and its alterations in human cancers for targeted therapy. Cell Signal.

[3] Zhou L, Jiang Y, Luo Q, Li L, Jia L (2019). Neddylation: a novel modulator of the tumor microenvironment. Mol Cancer.

[4] Mergner J, Schwechheimer C (2014). The NEDD8 modification pathway in plants. Front Plant Sci.

[5] Swatek KN, Komander D (2016). Ubiquitin modifications. Cell Res.

[6] Choi YS, Jeon YH, Ryu KS, Cheong C (2009). 60th residues of ubiquitin and Nedd8 are located out of E2-binding surfaces, but are important for K48 ubiquitin-linkage. FEBS Lett.

[7] Shen LN, Liu H, Dong C, Xirodimas D, Naismith JH, Hay RT (2005). Structural basis of NEDD8 ubiquitin discrimination by the deNEDDylating enzyme NEDP1. EMBO J.

[8] Reverter D, Wu K, Erdene TG, Pan ZQ, Wilkinson KD, Lima CD (2005). Structure of a complex between Nedd8 and the Ulp/Senp protease family member Den1. J Mol Biol.

[9] Walden H, Podgorski MS, Huang DT, Miller DW, Howard RJ, Minor DL, Holton JM, Schulman BA (2003). The structure of the APPBP1-UBA3-NEDD8-ATP complex reveals the basis for selective ubiquitin-like protein activation by an E1. Mol Cell.

[10] Schwechheimer C (2018). NEDD8-its role in the regulation of Cullin-RING ligases. Curr Opin Plant Biol.

[11] Yu Q, Jiang Y, Sun Y (2020). Anticancer drug discovery by targeting cullin neddylation. Acta Pharm Sin B.

[12] Santonico E (2020). Old and new concepts in ubiquitin and NEDD8 recognition. Biomolecules.

[13] Watson IR, Irwin MS, Ohh M (2011). NEDD8 pathways in cancer, Sine Quibus Non. Cancer Cell.

[14] Wang M, Medeiros BC, Erba HP, DeAngelo DJ, Giles FJ, Swords RT (2011). Targeting protein neddylation: a novel therapeutic strategy for the treatment of cancer. Expert Opin Ther Targets.

[15] Zhao Y, Morgan MA, Sun Y (2014). Targeting neddylation pathways to inactivate cullin-RING ligases for anticancer therapy. Antioxid Redox Signal.

[16] Baek K, Scott DC, Schulman BA (2020). NEDD8 and ubiquitin ligation by cullin-RING E3 ligases. Curr Opin Struct Biol.

[17] Li J, Zou J, Littlejohn R, Liu J, Su H (2020). Neddylation, an emerging mechanism regulating cardiac development and function. Front Physiol.

[18] Li L, Wang M, Yu G, Chen P, Li H, Wei D, Zhu J, Xie L, Jia H, Shi J (2014). Overactivated neddylation pathway as a therapeutic target in lung cancer. J Natl Cancer Inst.

[19] Yu J, Huang WL, Xu QG, Zhang L, Sun SH, Zhou WP, Yang F (2018). Overactivated neddylation pathway in human hepatocellular carcinoma. Cancer Med.

[20] Hua W, Li C, Yang Z, Li L, Jiang Y, Yu G, Zhu W, Liu Z, Duan S, Chu Y (2015). Suppression of glioblastoma by targeting the overactivated protein neddylation pathway. Neuro Oncol.

[21] Zhou S, Zhao X, Yang Z, Yang R, Chen C, Zhao K, Wang W, Ma Y, Zhang Q, Wang X (2019). Neddylation inhibition upregulates PD-L1 expression and enhances the efficacy of immune checkpoint blockade in glioblastoma. Int J Cancer.

[22] Xie P, Zhang M, He S, Lu K, Chen Y, Xing G, Lu Y, Liu P, Li Y, Wang S (2014). The covalent modifier Nedd8 is critical for the activation of Smurf1 ubiquitin ligase in tumorigenesis. Nat Commun.

[23] Gao Q, Yu GY, Shi JY, Li LH, Zhang WJ, Wang ZC, Yang LX, Duan M, Zhao H, Wang XY (2014). Neddylation pathway is up-regulated in human intrahepatic cholangiocarcinoma and serves as a potential therapeutic target. Oncotarget.

[24] Zhou Z, Song X, Wavelet CM, Wan Y (2020). Cullin 4-DCAF proteins in tumorigenesis. Adv Exp Med Biol.

[25] Bohnsack RN, Haas AL (2003). Conservation in the mechanism of Nedd8 activation by the human AppBp1-Uba3 heterodimer. J Biol Chem.

[26] Huang DT, Schulman BA (2005). Expression, purification, and characterization of the E1 for human NEDD8, the heterodimeric APPBP1-UBA3 complex. Methods Enzymol.

[27] Malik-Chaudhry HK, Gaieb Z, Saavedra A, Reyes M, Kung R, Le F, Morikis D, Liao J (2018). Dissecting distinct roles of NEDDylation E1 ligase heterodimer APPBP1 and UBA3 reveals potential evolution process for activation of ubiquitin-related pathways. Sci Rep.

[28] Chen Y, Neve R, Zheng H, Griffin W, Barger S, Mrak R (2014). Cycle on wheels: Is APP key to the AppBp1 pathway?. Austin Alzheimers Parkinsons Dis..

[29] Huang DT, Ayrault O, Hunt HW, Taherbhoy AM, Duda DM, Scott DC, Borg LA, Neale G, Murray PJ, Roussel MF, Schulman BA (2009). E2-RING expansion of the NEDD8 cascade confers specificity to cullin modification. Mol Cell.

[30] Lumpkin RJ, Ahmad AS, Blake R, Condon CJ, Komives EA (2020). The Mechanism of NEDD8 Activation of CUL5 Ubiquitin E3 Ligases. Mol Cell Proteomics.

[31] Zhou L, Zhu J, Chen W, Jiang Y, Hu T, Wang Y, Ye X, Zhan M, Ji C, Xu Z (2020). Induction of NEDD8-conjugating enzyme E2 UBE2F by platinum protects lung cancer cells from apoptosis and confers to platinum-insensitivity. Cell Death Dis.

[32] Zhang S, Sun Y (2020). Cullin RING Ligase 5 (CRL-5): neddylation activation and biological functions. Adv Exp Med Biol.

[33] Kamura T, Conrad MN, Yan Q, Conaway RC, Conaway JW (1999). The Rbx1 subunit of SCF and VHL E3 ubiquitin ligase activates Rub1 modification of cullins Cdc53 and Cul2. Genes Dev.

[34] Xie Y, Liu YK, Guo ZP, Guan H, Liu XD, Xie DF, Jiang YG, Ma T, Zhou PK (2020). RBX1 prompts degradation of EXO1 to limit the homologous recombination pathway of DNA double-strand break repair in G1 phase. Cell Death Differ.

[35] Cardote TAF, Gadd MS, Ciulli A (2017). Crystal structure of the Cul2-Rbx1-EloBC-VHL ubiquitin ligase complex. Structure.

[36] Kumar A, Shaha C (2018). RBX1-mediated ubiquitination of SESN2 promotes cell death upon prolonged mitochondrial damage in SH-SY5Y neuroblastoma cells. Mol Cell Biochem.

[37] Duan H, Wang Y, Aviram M, Swaroop M, Loo JA, Bian J, Tian Y, Mueller T, Bisgaier CL, Sun Y (1999). SAG, a novel zinc RING finger protein that protects cells from apoptosis induced by redox agents. Mol Cell Biol.

[38] Wang X, Wang X, Wang W, Zhang J, Wang J, Wang C, Lv M, Zuo T, Liu D, Zhang H (2015). Both Rbx1 and Rbx2 exhibit a functional role in the HIV-1 Vif-Cullin5 E3 ligase complex in vitro. Biochem Biophys Res Commun.

[39] Fairchild CL, Hino K, Han JS, Miltner AM, Peinado Allina G, Brown CE, Burns ME, La Torre A, Simó S (2018). RBX2 maintains final retinal cell position in a DAB1-dependent and -independent fashion. Development.

[40] Mathewson ND, Fujiwara H, Wu SR, Toubai T, Oravecz-Wilson K, Sun Y, Rossi C, Zajac C, Sun Y, Reddy P (2016). SAG/Rbx2-dependent neddylation regulates T-cell responses. Am J Pathol.

[41] Hino K, Simó S, Cooper JA (2018). Comparative analysis of cul5 and rbx2 expression in the developing and adult murine brain and their essentiality during mouse embryogenesis. Dev Dyn.

[42] Xiong X, Mathewson ND, Li H, Tan M, Fujiwara H, Li H, Reddy P, Sun Y (2018). SAG/RBX2 E3 ubiquitin ligase differentially regulates inflammatory responses of myeloid cell subsets. Front Immunol.

[43] Xirodimas DP, Saville MK, Bourdon JC, Hay RT, Lane DP (2004). Mdm2-mediated NEDD8 conjugation of p53 inhibits its transcriptional activity. Cell.

[44] Watson IR, Blanch A, Lin DC, Ohh M, Irwin MS (2006). Mdm2-mediated NEDD8 modification of TAp73 regulates its transactivation function. J Biol Chem.

[45] Dohmesen C, Koeppel M, Dobbelstein M (2008). Specific inhibition of Mdm2-mediated neddylation by Tip60. Cell Cycle.

[46] Oved S, Mosesson Y, Zwang Y, Santonico E, Shtiegman K, Marmor MD, Kochupurakkal BS, Katz M, Lavi S, Cesareni G, Yarden Y (2006). Conjugation to Nedd8 instigates ubiquitylation and down-regulation of activated receptor tyrosine kinases. J Biol Chem.

[47] Zuo W, Huang F, Chiang YJ, Li M, Du J, Ding Y, Zhang T, Lee HW, Jeong LS, Chen Y (2013). c-Cbl-mediated neddylation antagonizes ubiquitination and degradation of the TGF-β type II receptor. Mol Cell.

[48] Kong L, Wang B, Yang X, He B, Hao D, Yan L (2020). Integrin-associated molecules and signalling cross talking in osteoclast cytoskeleton regulation. J Cell Mol Med.

[49] Li X, Gong L, Gu H (2019). Regulation of immune system development and function by Cbl-mediated ubiquitination. Immunol Rev.

[50] Abida WM, Nikolaev A, Zhao W, Zhang W, Gu W (2007). FBXO11 promotes the neddylation of p53 and inhibits its transcriptional activity. J Biol Chem.

[51] Bornstein G, Ganoth D, Hershko A (2006). Regulation of neddylation and deneddylation of cullin1 in SCFSkp2 ubiquitin ligase by F-box protein and substrate. Proc Natl Acad Sci USA.

[52] Jansen S, van der Werf IM, Innes AM, Afenjar A, Agrawal PB, Anderson IJ, Atwal PS, van Binsbergen E, van den Boogaard MJ, Castiglia L (2019). De novo variants in FBXO11 cause a syndromic form of intellectual disability with behavioral problems and dysmorphisms. Eur J Hum Genet.

[53] Broemer M, Tenev T, Rigbolt KT, Hempel S, Blagoev B, Silke J, Ditzel M, Meier P (2010). Systematic in vivo RNAi analysis identifies IAPs as NEDD8-E3 ligases. Mol Cell.

[54] Kamada S (2013). Inhibitor of apoptosis proteins as E3 ligases for ubiquitin and NEDD8. Biomol Concepts.

[55] Akazawa Y, Guicciardi ME, Cazanave SC, Bronk SF, Werneburg NW, Kakisaka K, Nakao K, Gores GJ (2013). Degradation of cIAPs contributes to hepatocyte lipoapoptosis. Am J Physiol Gastrointest Liver Physiol.

[56] Rabut G, Le Dez G, Verma R, Makhnevych T, Knebel A, Kurz T, Boone C, Deshaies RJ, Peter M (2011). The TFIIH subunit Tfb3 regulates cullin neddylation. Mol Cell.

[57] Noguchi K, Okumura F, Takahashi N, Kataoka A, Kamiyama T, Todo S, Hatakeyama S (2011). TRIM40 promotes neddylation of IKKγ and is downregulated in gastrointestinal cancers. Carcinogenesis.

[58] Li T, Guan J, Huang Z, Hu X, Zheng X (2014). RNF168-mediated H2A neddylation antagonizes ubiquitylation of H2A and regulates DNA damage repair. J Cell Sci.

[59] Ma T, Chen Y, Zhang F, Yang CY, Wang S, Yu X (2013). RNF111-dependent neddylation activates DNA damage-induced ubiquitination. Mol Cell.

[60] Ho SR, Mahanic CS, Lee YJ, Lin WC (2014). RNF144A, an E3 ubiquitin ligase for DNA-PKcs, promotes apoptosis during DNA damage. Proc Natl Acad Sci U S A.

[61] Kurz T, Ozlü N, Rudolf F, O'Rourke SM, Luke B, Hofmann K, Hyman AA, Bowerman B, Peter M (2005). The conserved protein DCN-1/Dcn1p is required for cullin neddylation in *C. elegans* and *S. cerevisiae*. Nature.

[62] Kurz T, Chou YC, Willems AR, Meyer-Schaller N, Hecht ML, Tyers M, Peter M, Sicheri F (2008). Dcn1 functions as a scaffold-type E3 ligase for cullin neddylation. Mol Cell.

[63] Lin L, Chen L, Tran PT (2017). Fission yeast neddylation ligase Dcn1 facilitates cohesin cleavage and chromosome segregation at anaphase. Biol Open.

[64] Fang Y, Yu B, Liao G, Liu HM (2019). Targeting the DCN1-UBC12 protein-protein interaction: novel approaches and future directions. Future Med Chem.

[65] Rabut G, Peter M (2008). Function and regulation of protein neddylation. 'Protein modifications: beyond the usual suspects' review series. EMBO Rep.

[66] Bailly AP, Perrin A, Serrano-Macia M, Maghames C, Leidecker O, Trauchessec H, Martinez-Chantar ML, Gartner A, Xirodimas DP (2019). The balance between mono- and NEDD8-chains controlled by NEDP1 upon DNA damage is a regulatory module of the HSP70 ATPase activity. Cell Rep.

[67] Mendoza HM, Shen LN, Botting C, Lewis A, Chen J, Ink B, Hay RT (2003). NEDP1, a highly conserved cysteine protease that deNEDDylates cullins. J Biol Chem.

[68] Keuss MJ, Hjerpe R, Hsia O, Gourlay R, Burchmore R, Trost M, Kurz T (2019). Unanchored tri-NEDD8 inhibits PARP-1 to protect from oxidative stress-induced cell death. EMBO J.

[69] Guan J, Zheng X (2019). NEDDylation regulates RAD18 ubiquitination and localization in response to oxidative DNA damage. Biochem Biophys Res Commun.

[70] Lyapina S, Cope G, Shevchenko A, Serino G, Tsuge T, Zhou C, Wolf DA, Wei N, Shevchenko A, Deshaies RJ (2001). Promotion of NEDD-CUL1 conjugate cleavage by COP9 signalosome. Science.

[71] Min KW, Kwon MJ, Park HS, Park Y, Yoon SK, Yoon JB (2005). CAND1 enhances deneddylation of CUL1 by COP9 signalosome. Biochem Biophys Res Commun.

[72] Cavadini S, Fischer ES, Bunker RD, Potenza A, Lingaraju GM, Goldie KN, Mohamed WI, Faty M, Petzold G, Beckwith RE (2016). Cullin-RING ubiquitin E3 ligase regulation by the COP9 signalosome. Nature.

[73] Schlierf A, Altmann E, Quancard J, Jefferson AB, Assenberg R, Renatus M, Jones M, Hassiepen U, Schaefer M, Kiffe M (2016). Targeted inhibition of the COP9 signalosome for treatment of cancer. Nat Commun.

[74] Milic J, Tian Y, Bernhagen J (2019). Role of the COP9 Signalosome (CSN) in cardiovascular diseases. Biomolecules.

[75] Faull SV, Lau AMC, Martens C, Ahdash Z, Hansen K, Yebenes H, Schmidt C, Beuron F, Cronin NB, Morris EP, Politis A (2019). Structural basis of cullin 2 RING E3 ligase regulation by the COP9 signalosome. Nat Commun.

[76] Cornelius RJ, Yang CL, Ellison DH (2020). Hypertension-causing cullin 3 mutations disrupt COP9 signalosome binding. Am J Physiol Renal Physiol.

[77] Wu JT, Lin HC, Hu YC, Chien CT (2005). Neddylation and deneddylation regulate Cul1 and Cul3 protein accumulation. Nat Cell Biol.

[78] Yin L, Xue Y, Shang Q, Zhu H, Liu M, Liu Y, Hu Q (2019). Pharmaceutical inhibition of neddylation as promising treatments for various cancers. Curr Top Med Chem.

[79] Zhou Q, Li H, Li Y, Tan M, Fan S, Cao C, Meng F, Zhu L, Zhao L, Guan MX (2019). Inhibiting neddylation modification alters mitochondrial morphology and reprograms energy metabolism in cancer cells. JCI Insight.

[80] Petroski MD, Deshaies RJ (2005). Function and regulation of cullin-RING ubiquitin ligases. Nat Rev Mol Cell Biol.

[81] Huang DT, Miller DW, Mathew R, Cassell R, Holton JM, Roussel MF, Schulman BA (2004). A unique E1–E2 interaction required for optimal conjugation of the ubiquitin-like protein NEDD8. Nat Struct Mol Biol.

[82] Walden H, Podgorski MS, Schulman BA (2003). Insights into the ubiquitin transfer cascade from the structure of the activating enzyme for NEDD8. Nature.

[83] Huang DT, Paydar A, Zhuang M, Waddell MB, Holton JM, Schulman BA (2005). Structural basis for recruitment of Ubc12 by an E2 binding domain in NEDD8's E1. Mol Cell.

[84] Tokgöz Z, Siepmann TJ, Streich F, Kumar B, Klein JM, Haas AL (2012). E1–E2 interactions in ubiquitin and Nedd8 ligation pathways. J Biol Chem.

[85] Cukras S, Morffy N, Ohn T, Kee Y (2014). Inactivating UBE2M impacts the DNA damage response and genome integrity involving multiple cullin ligases. PLoS ONE.

[86] Zhang GC, Yu XN, Sun JL, Xiong J, Yang YJ, Jiang XM, Zhu JM (2020). UBE2M promotes cell proliferation via the β-catenin/cyclin D1 signaling in hepatocellular carcinoma. Aging (Albany NY).

[87] Li L, Kang J, Zhang W, Cai L, Wang S, Liang Y, Jiang Y, Liu X, Zhang Y, Ruan H (2019). Validation of NEDD8-conjugating enzyme UBC12 as a new therapeutic target in lung cancer. EBioMedicine.

[88] Wang S, Xian J, Li L, Jiang Y, Liu Y, Cai L, Hoffman RM, Jia L, Zhao H, Zhang Y (2020). NEDD8-conjugating enzyme UBC12 as a novel therapeutic target in esophageal squamous cell carcinoma. Signal Transduct Target Ther.

[89] Zhou W, Xu J, Li H, Xu M, Chen ZJ, Wei W, Pan Z, Sun Y (2017). Neddylation E2 UBE2F promotes the survival of lung cancer cells by activating CRL5 to degrade NOXA via the K11 linkage. Clin Cancer Res.

[90] Mao H, Tang Z, Li H, Sun B, Tan M, Fan S, Zhu Y, Sun Y (2019). Neddylation inhibitor MLN4924 suppresses cilia formation by modulating AKT1. Protein Cell.

[91] Wu MH, Lee CY, Huang TJ, Huang KY, Tang CH, Liu SH, Kuo KL, Kuan FC, Lin WC, Shi CS (2018). MLN4924, a protein neddylation inhibitor, suppresses the growth of human chondrosarcoma through inhibiting cell proliferation and inducing endoplasmic reticulum stress-related apoptosis. Int J Mol Sci.

[92] Soucy TA, Smith PG, Milhollen MA, Berger AJ, Gavin JM, Adhikari S, Brownell JE, Burke KE, Cardin DP, Critchley S (2009). An inhibitor of NEDD8-activating enzyme as a new approach to treat cancer. Nature.

[93] Soucy TA, Smith PG, Rolfe M (2009). Targeting NEDD8-activated cullin-RING ligases for the treatment of cancer. Clin Cancer Res.

[94] Brownell JE, Sintchak MD, Gavin JM, Liao H, Bruzzese FJ, Bump NJ, Soucy TA, Milhollen MA, Yang X, Burkhardt AL (2010). Substrate-assisted inhibition of ubiquitin-like protein-activating enzymes: the NEDD8 E1 inhibitor MLN4924 forms a NEDD8-AMP mimetic in situ. Mol Cell.

[95] Swords RT, Kelly KR, Smith PG, Garnsey JJ, Mahalingam D, Medina E, Oberheu K, Padmanabhan S, O'Dwyer M, Nawrocki ST (2010). Inhibition of NEDD8-activating enzyme: a novel approach for the treatment of acute myeloid leukemia. Blood.

[96] Zhou W, Xu J, Tan M, Li H, Li H, Wei W, Sun Y (2018). UBE2M is a stress-inducible dual E2 for neddylation and ubiquitylation that promotes targeted degradation of UBE2F. Mol Cell.

[97] Duncan K, Schäfer G, Vava A, Parker MI, Zerbini LF (2012). Targeting neddylation in cancer therapy. Future Oncol.

[98] Zhang Y, Shi CC, Zhang HP, Li GQ, Li SS (2016). MLN4924 suppresses neddylation and induces cell cycle arrest, senescence, and apoptosis in human osteosarcoma. Oncotarget.

[99] Heo MJ, Kang SH, Kim YS, Lee JM, Yu J, Kim HR, Lim H, Kim KM, Jung J, Jeong LS (2020). UBC12-mediated SREBP-1 neddylation worsens metastatic tumor prognosis. Int J Cancer.

[100] Ba C, Ni X, Yu J, Zou G, Zhu H (2020). Ubiquitin conjugating enzyme E2 M promotes apoptosis in osteoarthritis chondrocytes via Wnt/β-catenin signaling. Biochem Biophys Res Commun.

[101] Zhao B, Gao C, Shi D, Mao J, Zhao J, Guo L, Guo J, Jiao Z (2019). Knockdown of Nedd8-conjugating enzyme UBE2M suppresses the proliferation and induces the apoptosis of intrahepatic cholangiocarcinoma cells. Oncol Rep.

[102] Chen F, Zhang Y, Varambally S, Creighton CJ (2019). Molecular correlates of metastasis by systematic pan-cancer analysis across the cancer genome Atlas. Mol Cancer Res.

[103] Jiang Y, Cheng W, Li L, Zhou L, Liang Y, Zhang W, Chen W, Wang S, Zhao H, Chen G (2020). Effective targeting of the ubiquitin-like modifier NEDD8 for lung adenocarcinoma treatment. Cell Biol Toxicol.

[104] Tian DW, Wu ZL, Jiang LM, Gao J, Wu CL, Hu HL (2019). Neural precursor cell expressed, developmentally downregulated 8 promotes tumor progression and predicts poor prognosis of patients with bladder cancer. Cancer Sci.

[105] Lu G, Yi J, Gubas A, Wang YT, Wu Y, Ren Y, Wu M, Shi Y, Ouyang C, Tan HWS (2019). Suppression of autophagy during mitosis via CUL4-RING ubiquitin ligases-mediated WIPI2 polyubiquitination and proteasomal degradation. Autophagy.

[106] Jia L, Li H, Sun Y (2011). Induction of p21-dependent senescence by an NAE inhibitor, MLN4924, as a mechanism of growth suppression. Neoplasia.

[107] Lin JJ, Milhollen MA, Smith PG, Narayanan U, Dutta A (2010). NEDD8-targeting drug MLN4924 elicits DNA rereplication by stabilizing Cdt1 in S phase, triggering checkpoint activation, apoptosis, and senescence in cancer cells. Cancer Res.

[108] Mackintosh C, García-Domínguez DJ, Ordóñez JL, Ginel-Picardo A, Smith PG, Sacristán MP, de Álava E (2013). WEE1 accumulation and deregulation of S-phase proteins mediate MLN4924 potent inhibitory effect on Ewing sarcoma cells. Oncogene.

[109] Brown JS, Lukashchuk N, Sczaniecka-Clift M, Britton S, le Sage C, Calsou P, Beli P, Galanty Y, Jackson SP (2015). Neddylation promotes ubiquitylation and release of Ku from DNA-damage sites. Cell Rep.

[110] Jimeno S, Fernández-Ávila MJ, Cruz-García A, Cepeda-García C, Gómez-Cabello D, Huertas P (2015). Neddylation inhibits CtIP-mediated resection and regulates DNA double strand break repair pathway choice. Nucleic Acids Res.

[111] Wang X, Zhang W, Yan Z, Liang Y, Li L, Yu X, Feng Y, Fu S, Zhang Y, Zhao H (2016). Radiosensitization by the investigational NEDD8-activating enzyme inhibitor MLN4924 (pevonedistat) in hormone-resistant prostate cancer cells. Oncotarget.

[112] Zhao Y, Sun Y (2013). Cullin-RING Ligases as attractive anti-cancer targets. Curr Pharm Des.

[113] Soucy TA, Dick LR, Smith PG, Milhollen MA, Brownell JE (2010). The NEDD8 conjugation pathway and its relevance in cancer biology and therapy. Genes Cancer.

[114] Ying J, Zhang M, Qiu X, Lu Y (2018). Targeting the neddylation pathway in cells as a potential therapeutic approach for diseases. Cancer Chemother Pharmacol.

[115] Duda DM, Borg LA, Scott DC, Hunt HW, Hammel M, Schulman BA (2008). Structural insights into NEDD8 activation of cullin-RING ligases: conformational control of conjugation. Cell.

[116] Xu B, Deng Y, Bi R, Guo H, Shu C, Shah NK, Chang J, Liu G, Du Y, Wei W, Wang C (2018). A first-in-class inhibitor, MLN4924 (pevonedistat), induces cell-cycle arrest, senescence, and apoptosis in human renal cell carcinoma by suppressing UBE2M-dependent neddylation modification. Cancer Chemother Pharmacol.

[117] Lin S, Shang Z, Li S, Gao P, Zhang Y, Hou S, Qin P, Dong Z, Hu T, Chen P (2018). Neddylation inhibitor MLN4924 induces G(2) cell cycle arrest, DNA damage and sensitizes esophageal squamous cell carcinoma cells to cisplatin. Oncol Lett.

[118] Yao WT, Wu JF, Yu GY, Wang R, Wang K, Li LH, Chen P, Jiang YN, Cheng H, Lee HW (2014). Suppression of tumor angiogenesis by targeting the protein neddylation pathway. Cell Death Dis.

[119] Tong S, Si Y, Yu H, Zhang L, Xie P, Jiang W (2017). MLN4924 (Pevonedistat), a protein neddylation inhibitor, suppresses proliferation and migration of human clear cell renal cell carcinoma. Sci Rep.

[120] Milhollen MA, Thomas MP, Narayanan U, Traore T, Riceberg J, Amidon BS, Bence NF, Bolen JB, Brownell J, Dick LR (2012). Treatment-emergent mutations in NAEβ confer resistance to the NEDD8-activating enzyme inhibitor MLN4924. Cancer Cell.

[121] Xu GW, Toth JI, da Silva SR, Paiva SL, Lukkarila JL, Hurren R, Maclean N, Sukhai MA, Bhattacharjee RN, Goard CA (2014). Mutations in UBA3 confer resistance to the NEDD8-activating enzyme inhibitor MLN4924 in human leukemic cells. PLoS ONE.

[122] Ho IL, Kuo KL, Liu SH, Chang HC, Hsieh JT, Wu JT, Chiang CK, Lin WC, Tsai YC, Chou CT (2015). MLN4924 synergistically enhances cisplatin-induced cytotoxicity via JNK and Bcl-xL pathways in human urothelial carcinoma. Sci Rep.

[123] Zhou H, Lu J, Yang CY, Sun Y, Wang S (2020). Targeting DCN1-UBC12 protein–protein interaction for regulation of neddylation pathway. Adv Exp Med Biol.

[124] Zhou H, Lu J, Liu L, Bernard D, Yang CY, Fernandez-Salas E, Chinnaswamy K, Layton S, Stuckey J, Yu Q (2017). A potent small-molecule inhibitor of the DCN1-UBC12 interaction that selectively blocks cullin 3 neddylation. Nat Commun.

[125] Zhou H, Zhou W, Zhou B, Liu L, Chern TR, Chinnaswamy K, Lu J, Bernard D, Yang CY, Li S (2018). High-Affinity Peptidomimetic Inhibitors of the DCN1-UBC12 Protein-Protein Interaction. J Med Chem.

[126] Scott DC, Monda JK, Bennett EJ, Harper JW, Schulman BA (2011). N-terminal acetylation acts as an avidity enhancer within an interconnected multiprotein complex. Science.

[127] Scott DC, Monda JK, Grace CR, Duda DM, Kriwacki RW, Kurz T, Schulman BA (2010). A dual E3 mechanism for Rub1 ligation to Cdc53. Mol Cell.

[128] Scott DC, Rhee DY, Duda DM, Kelsall IR, Olszewski JL, Paulo JA, de Jong A, Ovaa H, Alpi AF, Harper JW, Schulman BA (2016). Two distinct types of E3 ligases work in unison to regulate substrate ubiquitylation. Cell.

[129] Scott DC, Hammill JT, Min J, Rhee DY, Connelly M, Sviderskiy VO, Bhasin D, Chen Y, Ong SS, Chai SC (2017). Blocking an N-terminal acetylation-dependent protein interaction inhibits an E3 ligase. Nat Chem Biol.

[130] Hammill JT, Bhasin D, Scott DC, Min J, Chen Y, Lu Y, Yang L, Kim HS, Connelly MC, Hammill C (2018). Discovery of an orally bioavailable inhibitor of defective in cullin neddylation 1 (DCN1)-mediated cullin neddylation. J Med Chem.

[131] Hammill JT, Scott DC, Min J, Connelly MC, Holbrook G, Zhu F, Matheny A, Yang L, Singh B, Schulman BA, Guy RK (2018). Piperidinyl ureas chemically control defective in cullin neddylation 1 (DCN1)-mediated cullin neddylation. J Med Chem.

[132] Zhou W, Ma L, Ding L, Guo Q, He Z, Yang J, Qiao H, Li L, Yang J, Yu S (2019). Potent 5-cyano-6-phenyl-pyrimidin-based derivatives targeting DCN1-UBE2M interaction. J Med Chem.

[133] Kim HS, Hammill JT, Scott DC, Chen Y, Min J, Rector J, Singh B, Schulman BA, Guy RK (2019). Discovery of novel pyrazolo-pyridone DCN1 inhibitors controlling cullin neddylation. J Med Chem.

[134] Wang S, Zhao L, Shi XJ, Ding L, Yang L, Wang ZZ, Shen D, Tang K, Li XJ, Mamun M (2019). Development of highly potent, selective, and cellular active triazolo[1,5- a]pyrimidine-based inhibitors targeting the DCN1-UBC12 protein–protein interaction. J Med Chem.

[135] Shedden K, Taylor JM, Enkemann SA, Tsao MS, Yeatman TJ, Gerald WL, Eschrich S, Jurisica I, Giordano TJ, Misek DE (2008). Gene expression-based survival prediction in lung adenocarcinoma: a multi-site, blinded validation study. Nat Med.

[136] Hou J, Aerts J, den Hamer B, van Ijcken W, den Bakker M, Riegman P, van der Leest C, van der Spek P, Foekens JA, Hoogsteden HC (2010). Gene expression-based classification of non-small cell lung carcinomas and survival prediction. PLoS ONE.

